# Vernon Galbray, or the Empiric

**Published:** 1876-01

**Authors:** 


*Vernon Galbray, or the Empiric.*—The history of a Quack Dentist. London, E. T. Whitfield, 178 Strand.

This is an amusing tale, professing to be an *expose* of the disgraceful frauds practiced upon the public by the charlatans who call themselves dentists, and the author promises to substantiate every charge against what he supposes some may consider "an exaggerated picture." That people will allow themselves to be the subjects of humbug notwithstanding repeated warnings, is patent every day of the year, and such a publication as this, although perhaps published with the best of objects, will avail but little in correcting the evil.

